# Cross-sectional anatomy, computed tomography, and magnetic resonance imaging of the banded houndshark (*Triakis scyllium*)

**DOI:** 10.1038/s41598-020-80823-y

**Published:** 2021-01-13

**Authors:** Sang Wha Kim, Adams Hei Long Yuen, Cherry Tsz Ching Poon, Joon Oh Hwang, Chang Jun Lee, Moon-Kwan Oh, Ki Tae Kim, Hyoun Joong Kim, Sib Sankar Giri, Sang Guen Kim, Jun Kwon, Sung Bin Lee, Min Cheol Choi, Se Chang Park

**Affiliations:** 1 College of Veterinary Medicine and the Research Institute for Veterinary Science, Seoul National University, Seoul, 08826 Republic of Korea; 2 Department of Surgery, Queen Mary Hospital, Pokfulam, Hong Kong Special Administrative Region China; 3 Hyemin Animal Hospital, Seoul, 06239 Republic of Korea; 4 HDX Corporation, Seoul, 03162 Republic of Korea

**Keywords:** Marine biology, Anatomy, Ichthyology

## Abstract

Due to their important phylogenetic position among extant vertebrates, sharks are an invaluable group in evolutionary developmental biology studies. A thorough understanding of shark anatomy is essential to facilitate these studies and documentation of this iconic taxon. With the increasing availability of cross-sectional imaging techniques, the complicated anatomy of both cartilaginous and soft tissues can be analyzed non-invasively, quickly, and accurately. The aim of this study is to provide a detailed anatomical description of the normal banded houndshark (*Triakis scyllium*) using computed tomography (CT) and magnetic resonance imaging (MRI) along with cryosection images. Three banded houndsharks were scanned using a 64-detector row spiral CT scanner and a 3 T MRI scanner. All images were digitally stored and assessed using open-source Digital Imaging and Communications in Medicine viewer software in the transverse, sagittal, and dorsal dimensions. The banded houndshark cadavers were then cryosectioned at approximately 1-cm intervals. Corresponding transverse cryosection images were chosen to identify the best anatomical correlations for transverse CT and MRI images. The resulting images provided excellent detail of the major anatomical structures of the banded houndshark. The illustrations in the present study could be considered as a useful reference for interpretation of normal and pathological imaging studies of sharks.

## Introduction

The past half-century has seen a sharp decline in the population of sharks due to indiscriminate fishing, and they are now facing a severe extinction crisis^1–4^. Sharks are apex predators and keystone species, playing an important role in maintaining the marine ecosystem. Thus, extinction of sharks in certain areas is expected to lead to marine food chain collapses, which could result in a sharp reduction in the marine food resources available for human beings^5–7^. As a result, the importance of protecting endangered shark species has been recognized worldwide, and efforts to study these species to understand and protect them are being made by various groups, including aquariums, fishery industries, and biologists^7–12^.

With the increasing availability of cross-sectional imaging techniques such as computed tomography (CT) and magnetic resonance imaging (MRI), the complicated and sophisticated anatomy of both skeletal and soft tissues can be analyzed non-invasively, quickly, and accurately^13–16^. CT and MRI have often been used not only for humans but also for various animal species^13,17–19^. Some of these studies were conducted on sharks and involved diverse fields such as physiology, anatomy, developmental biology, archaeology, and evolutionary biology^9,20–28^.

Although a wide variety of studies have been performed on sharks^29^, official publications describing the basic anatomy of sharks particularly in CT and/or MRI evaluations are currently unavailable. Accurate evaluation of CT and MRI images requires precise knowledge of the anatomy and physiology of the animal. In this regard, the development of accurate atlases for certain species by comparing CT and MRI images with actual cryosections is one of the primary steps that should be established by researchers and veterinarians^30^. Normal atlases of CT and MRI findings in various animals have already been set up in order to facilitate accurate image analysis^30–38^. In the same context, precise atlases of CT and MRI findings in shark species should also be established.

The banded houndshark (*Triakis scyllium*) is a relatively small-sized shark that is easily found in Korean water. They inhabit the Northwest Pacific Ocean and are classified as the “least concern” group by the International Union for Conservation of Nature (IUCN) red list^4,39^. The purpose of this study is to set up a detailed atlas of the banded houndshark by comparing CT and MRI images with actual cryosections. To the best of our knowledge, this is the first study reporting delicate comparison of CT, MRI, and cryosection findings for shark anatomy.

## Results

In the present study, CT and MRI allowed identification of a broad selection of anatomical structures and correlated well with the corresponding cryosections of the banded houndshark. Clinically relevant anatomical structures are labeled in Figs. 1, 2, 3, 4, 5, 6, 7, 8, 9, 10, 11, 12, 13, 14, 15, 16, 17, 18, 19, 20, 21, 22, 23. Figures 1, 2, 3, 4, 5, 6, 7, 8, 9, 10, 11, 12, 13, 14 (transverse images), Figs. 15, 16, 17 (sagittal images), and Figs. 18, 19, 20, 21, 22, 23 (dorsal images) present the findings in cranial to caudal, right to left, and dorsal to ventral progression, respectively. Three-dimensional (3D) reconstructed images of CT and MRI data sets showing major skeletons and organs of the banded houndshark were presented in Fig. 24. Transverse images of MRI and CT are shown continuously in Supplementary Video S1 and Supplementary Video S2, respectively, to make up for the shortcomings of discontinuous cross-sectional images.

**Figure 1:**
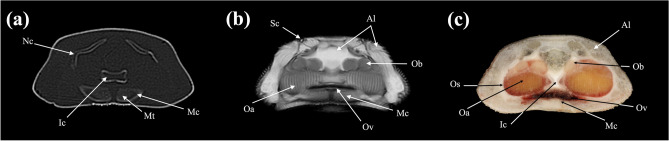
Transverse (**a**) CT, (**b**) MR, and (**c**) cryosection images of a banded houndshark (*Triakis scyllium*) at level 1 of Fig. 25. *Al* Ampullae of Lorenzini, *Ic* Internasal cartilage, *Mc* Meckel’s cartilage, *Mt* Mandibular teeth, *Nc* Nasal capsule cartilage, *Oa* Olfactory lamellae, *Ob* Olfactory bulb, *Os* Olfactory sac, *Ov* Oral cavity, *Sc* Supraorbital canal.

**Figure 2:**
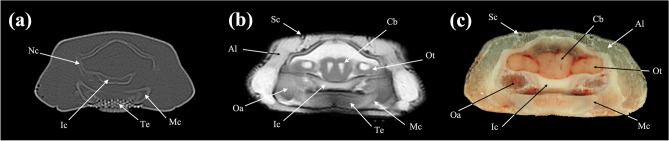
Transverse (**a**) CT, (**b**) MR, and (**c**) cryosection images of a banded houndshark (*Triakis scyllium*) at level 2 of Fig. 25. *Al* Ampullae of Lorenzini, *Cb* Cerebrum, *Ic* Internasal cartilage, *Mc* Meckel’s cartilage, *Te* Teeth, *Nc* Nasal capsule cartilage, *Oa* Olfactory lamellae, *Ot* Olfactory tract, *Sc* Supraorbital canal.

**Figure 3:**
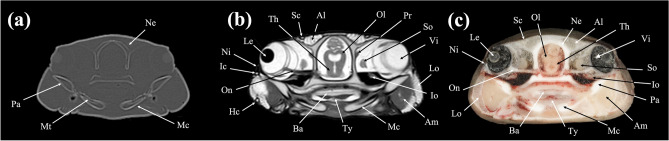
Transverse (**a**) CT, (**b**) MR, and (**c**) cryosection images of a banded houndshark (*Triakis scyllium*) at level 3 of Fig. 25. *Al* Ampullae of Lorenzini, *Am* Adductor mandibularis, *Ba* Basihyal cartilage, *Hc* Hyomandibular canal, *Ic* Infraorbital canal, *Io* Inferior obliquus, *Le* Lens, *Mc* Meckel’s cartilage, *Mt* Mandibular teeth, *Ne* Neurocranium, *Ni* Nictitating fold, *Ol* Optic lobe, *On* Optic nerve, *Pa* Palatoquadrate, *Pr* Posterior rectus, *Sc* Supraorbital canal, *So* Superior obliquus, *Th* Thalamus, *Ty* Thyroid, *Vi* Vitreous humor.

**Figure 4:**
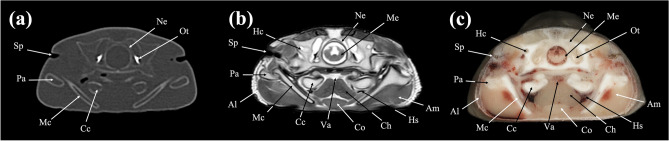
Transverse (**a**) CT, (**b**) MR, and (**c**) cryosection images of a banded houndshark (*Triakis scyllium*) at level 4 of Fig. 25. *Al* Ampullae of Lorenzini, *Am* Adductor mandibularis, *Cc* Ceratohyal cartilage, *Ch* Coracohyoideus, *Co* Coracomandibularis, *Hc* Horizontal semicircular canal, *Hs* Hyoidean sinus, *Me* Medulla Oblongata, *Mc* Meckel’s cartilage, *Ne* Neurocranium, *Ot* Otolith, *Pa* Palatoquadrate, *Sp* Spiracle, *Va* Ventral aorta.

**Figure 5:**
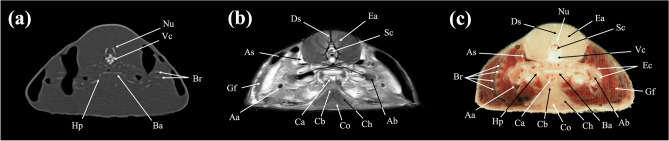
Transverse (**a**) CT, (**b**) MR, and (**c**) cryosection images of a banded houndshark (*Triakis scyllium*) at level 5 of Fig. 25. *Aa* Afferent branchial artery, *Ab* Adductor branchial, *As* Anterior cardinal sinus, *Ba* Basibranchial, *Br* Branchial rays, *Ca* Conus arteriosus, *Cb* Coracobranchialis, *Ch* Coracohyoideus, *Co* Coracomandibularis, *Ds* Dorsal skeletogenous septum, *Ea* Epaxial, *Ec* Epibranchial and ceratobranchial cartilage, *Gf* Gill filament, *Hp* Hypobranchial cartilage, *Nu* Neural arch, *Vc* Vertebral centrum.

**Figure 6:**
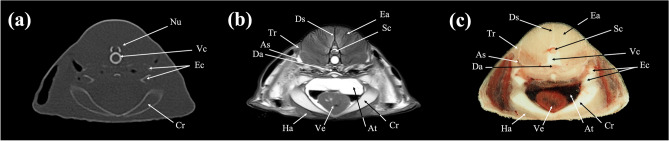
Transverse (**a**) CT, (**b**) MR, and (**c**) cryosection images of a banded houndshark (*Triakis scyllium*) at level 6 of Fig. 25. *As* Anterior cardinal sinus, *At* Atrium, *Cr* Coracoid bar, *Da* Dorsal aorta, *Ds* Dorsal median septum, *Ea* Epaxial, *Ec* Epibranchial and ceratobranchial cartilage, *Ha* Hypaxial, *Sc* Spinal cord, *Tr* Trapezius, *Ve* Ventricle, *Vc* Vertebral centrum.

**Figure 7:**
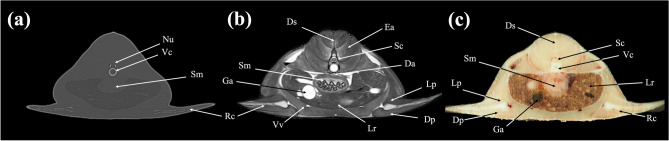
Transverse (**a**) CT, (**b**) MR, and (**c**) cryosection images of a banded houndshark (*Triakis scyllium*) at level 7 of Fig. 25. *Da* Dorsal aorta, *Dp* Depressor pectoralis, *Ds* Dorsal median septum, *Ea* Epaxial, *Ga* Gall bladder, *Ha* Hypaxial, *Lp* Levator pectoralis internus, *Nu* Neural arch, *Lr* Liver, *Rc* Radial cartilage, *Sc* Spinal cord, *Sm* Stomach, *Vc* Vertebral centrum, *Vv* Ventral abdominal vein.

**Figure 8:**
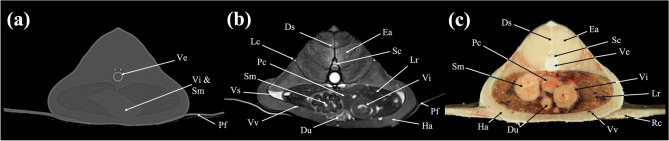
Transverse (**a**) CT, (**b**) MR, and (**c**) cryosection images of a banded houndshark (*Triakis scyllium*) at level 8 of Fig. 25. *Ds* Dorsal median septum, *Du* Duodenum, *Ea* Epaxial, *Ha* Hypaxial, *Id* Inclinator dorsalis, *Kd* Kidney, *Lc* Lateral line canal, *Lr* Liver, *Pc* Pancreas, *Pf* Pectoral fin, *Rc* Radial cartilage, *Sc* Spinal cord, *Sm* Stomach, *Ve* Vertebra, *Vi* Valvular intestine, *Vs* Visceral cavity, *Vv* Ventral abdominal vein.

**Figure 9:**
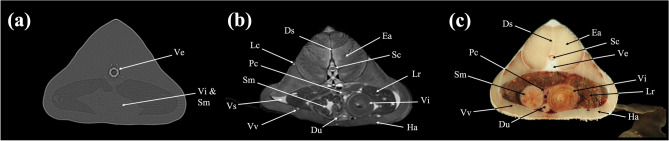
Transverse (**a**) CT, (**b**) MR, and (**c**) cryosection images of a banded houndshark (*Triakis scyllium*) at level 9 of Fig. 25. *Ds* Dorsal median septum, *Du* Duodenum, *Ea* Epaxial, *Ha* Hypaxial, *Id* Inclinator dorsalis, *Kd* Kidney, *Lc* Lateral line canal, *Lr* Liver, *Pc* Pancreas, *Rc* Radial cartilage, *Sc* Spinal cord, *Sm* Stomach, *Ve* Vertebra, *Vi* Valvular intestine, *Vs* Visceral cavity, *Vv* Ventral abdominal vein.

**Figure 10:**
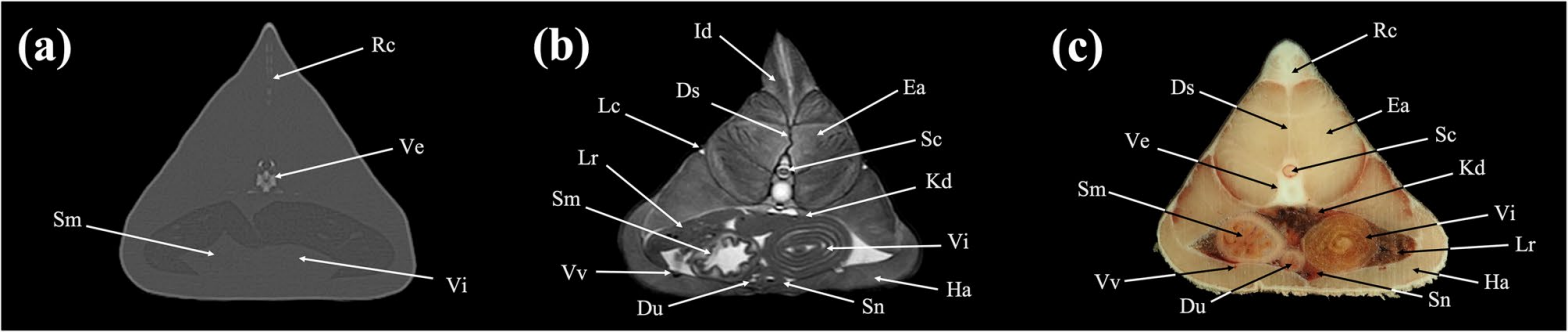
Transverse (**a**) CT, (**b**) MR, and (**c**) cryosection images of a banded houndshark (*Triakis scyllium*) at level 10 of Fig. 25. *Ds* Dorsal median septum, *Du* Duodenum, *Ea* Epaxial, *Ha* Hypaxial, *Id* Inclinator dorsalis, *Kd* Kidney, *Lc* Lateral line canal, *Lr* Liver, *Rc* Radial cartilage, *Sc* Spinal cord, *Sm* Stomach, *Sn* Spleen, *Ve* Vertebra, *Vi* Valvular intestine, *Vv* Ventral abdominal vein.

**Figure 11:**
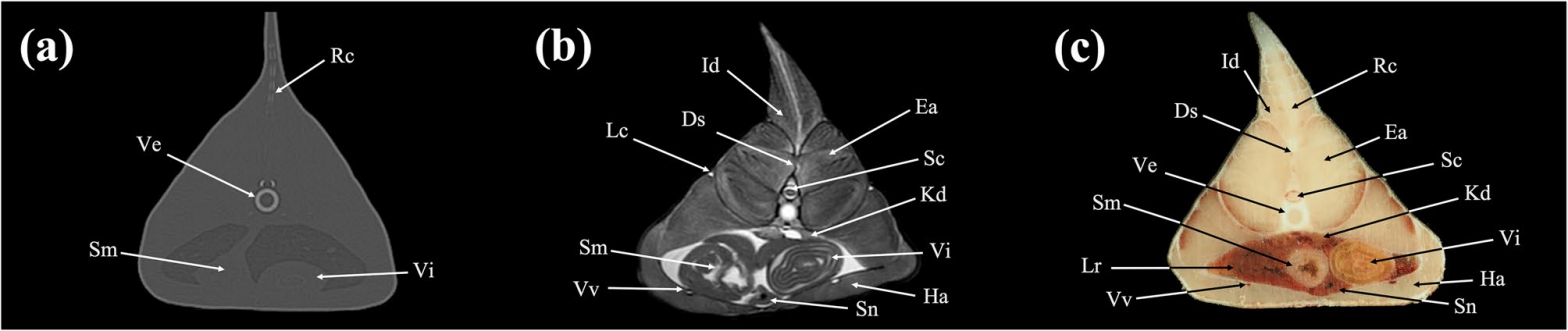
Transverse (**a**) CT, (**b**) MR, and (**c**) cryosection images of a banded houndshark (*Triakis scyllium*) at level 11 of Fig. 25. *Ds* Dorsal median septum, *Ea* Epaxial, *Ha* Hypaxial, *Id* Inclinator dorsalis, *Kd* Kidney, *Lc* Lateral line canal, *Lr* Liver, *Rc* Radial cartilage, *Sc* Spinal cord, *Sm* Stomach, *Sn* Spleen, *Ve* Vertebra, *Vi* Valvular intestine, *Vv* Ventral abdominal vein.

**Figure 12:**
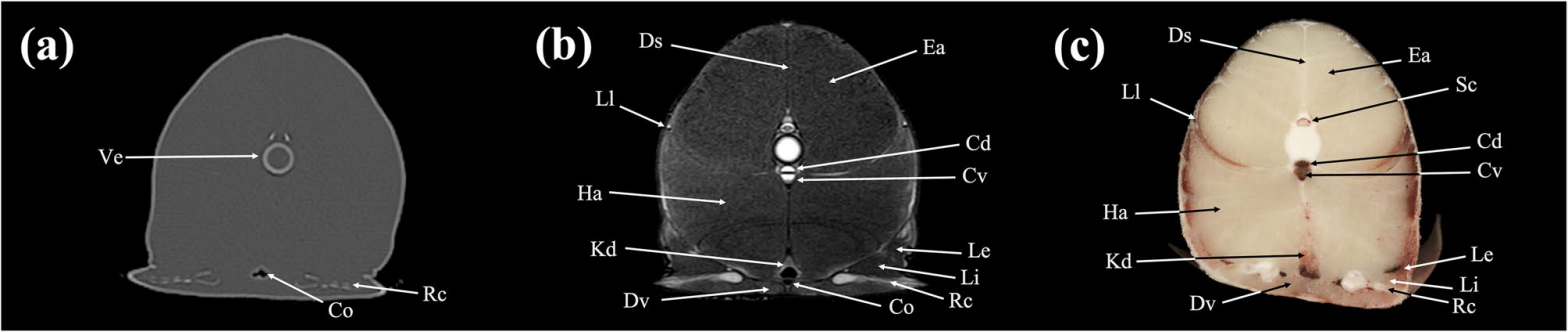
Transverse (**a**) CT, (**b**) MR, and (**c**) cryosection images of a banded houndshark (*Triakis scyllium*) at level 12 of Fig. 25. *Cd* Caudal artery, *Co* cloaca, *Cv* Caudal vein, *Ds* Dorsal skeletogenous septum, *Dv* Depressor ventralis, *Ea* Epaxial, *Ha* Hypaxial, *Kd* Kidney, *Le* Levator ventralis externus, *Li* Levator ventralis internus, *Ll* Lateral line canal, *Rc* Radial cartilage, *Sc* Spinal cord.

**Figure 13:**
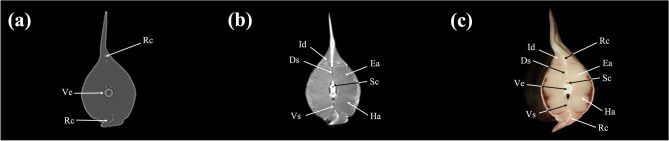
Transverse (**a**) CT, (**b**) MR, and (**c**) cryosection images of a banded houndshark (*Triakis scyllium*) at level 13 of Fig. 25. *Ds* Dorsal skeletogenous septum, *Ea* Epaxial, *Ha* Hypaxial, *Id* Inclinator dorsalis, *Rc* Radial cartilage, *Sc* Spinal cord, *Ve* Vertebra, *Vs* Ventral skeletogenous septum.

**Figure 14:**
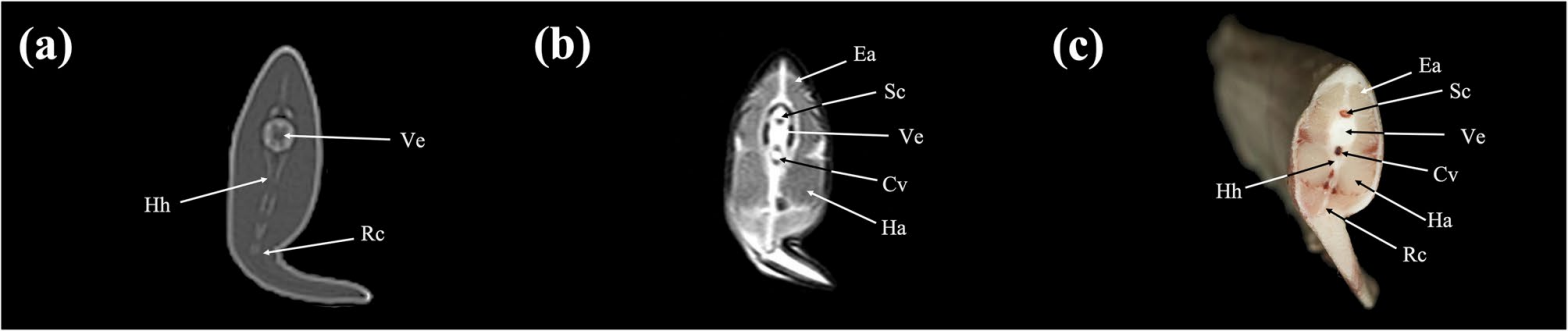
Transverse (**a**) CT, (**b**) MR, and (**c**) cryosection images of a banded houndshark (*Triakis scyllium*) at level 14 of Fig. 25. *Cv* Caudal vessel, *Ea* Epaxial, *Ha* Hypaxial, *Hh* Haemal arch, *Rc* Radial cartilage, *Sc* Spinal cord, *Ve* Vertebra.

**Figure 15:**
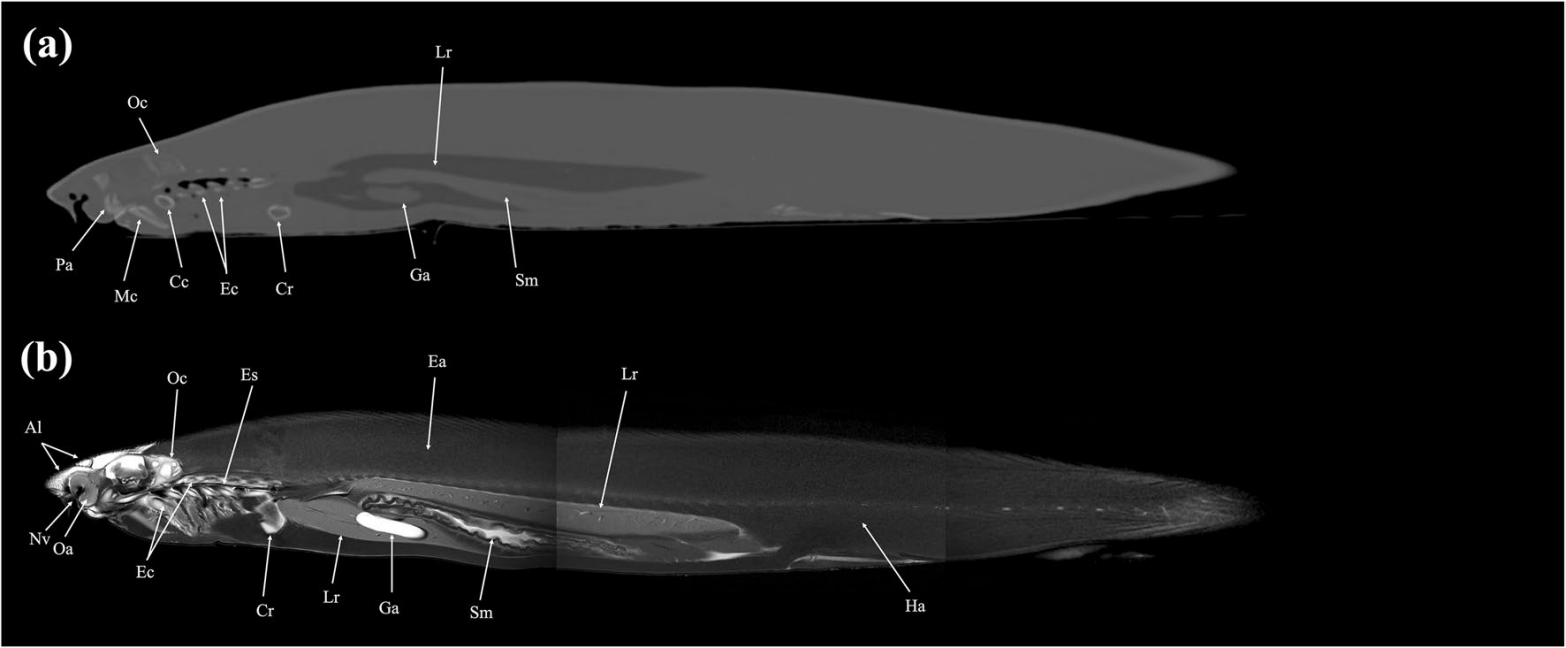
Sagittal (**a**) CT and (**b**) MR images of a banded houndshark (*Triakis scyllium*) at level 15 of Fig. 25. *Al* Ampullae of Lorenzini, *Cc* Ceratohyal cartilage, *Cr* Coracoid bar, *Ec* Epibranchial and ceratobranchial cartilage, *Es* Esophagus, *Ga* Gall bladder, *Mc* Meckel’s cartilage, *Oa* Olfactory lamellae, *Oc* Otic capsule, *Pa* Palatoquadrate, *Sm* Stomach, *Nv* Nasal cavity.

**Figure 16:**
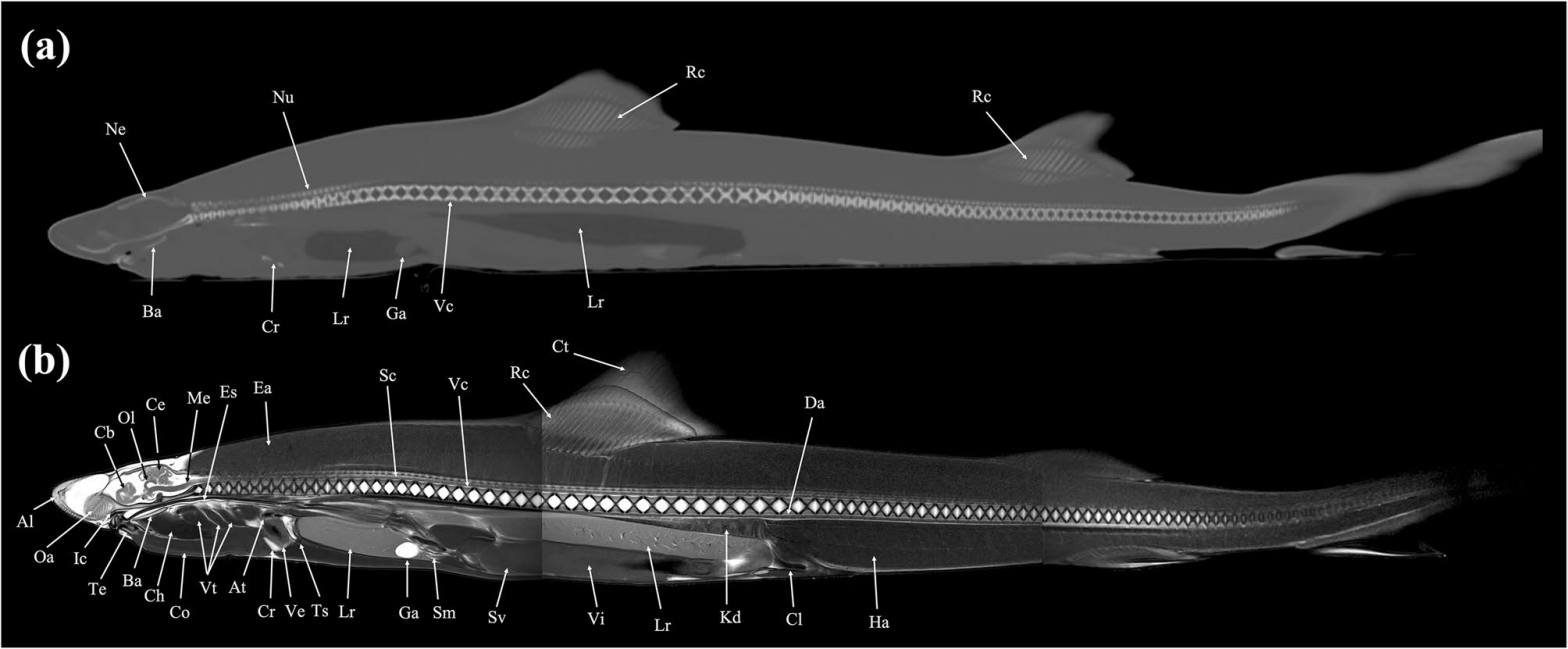
Mid-sagittal (**a**) CT and (**b**) MR images of a banded houndshark (*Triakis scyllium*) at level 16 of Fig. 25. *Al* Ampullae of Lorenzini, *At* Atrium, *Ba* Basihyal cartilage, *Cb* Cerebrum, *Ce* Cerebellum, *Cl* cloaca, *Co* Coracomandibularis, *Cr* Coracoid bar, *Ch* Coracohyoideus, *Ct* Ceratotrichia, *Da* Dorsal aorta, *Ea* Epaxial, *Es* Esophagus, *Ga* Gall bladder, *Ha* Hypaxial, *Ic* Internasal cartilage, *Kd* Kidney, *Lr* Liver, *Me* Medulla oblongata, *Ne* Neurocranium, *Nu* Neural arch, *Oa* Olfactory lamellae, *Ol* Optic lobe, *Rc* Radial cartilage, *Sc* Spinal cord, *Te* Teeth, *Ts* Transverse septum, *Vi* Valvular intestine, *Ve* Ventricle, *Vt* Ventral constrictors, *Vc* Vertebral centrum, *Sm* Stomach, *Sv* Spiral valve.

**Figure 17:**
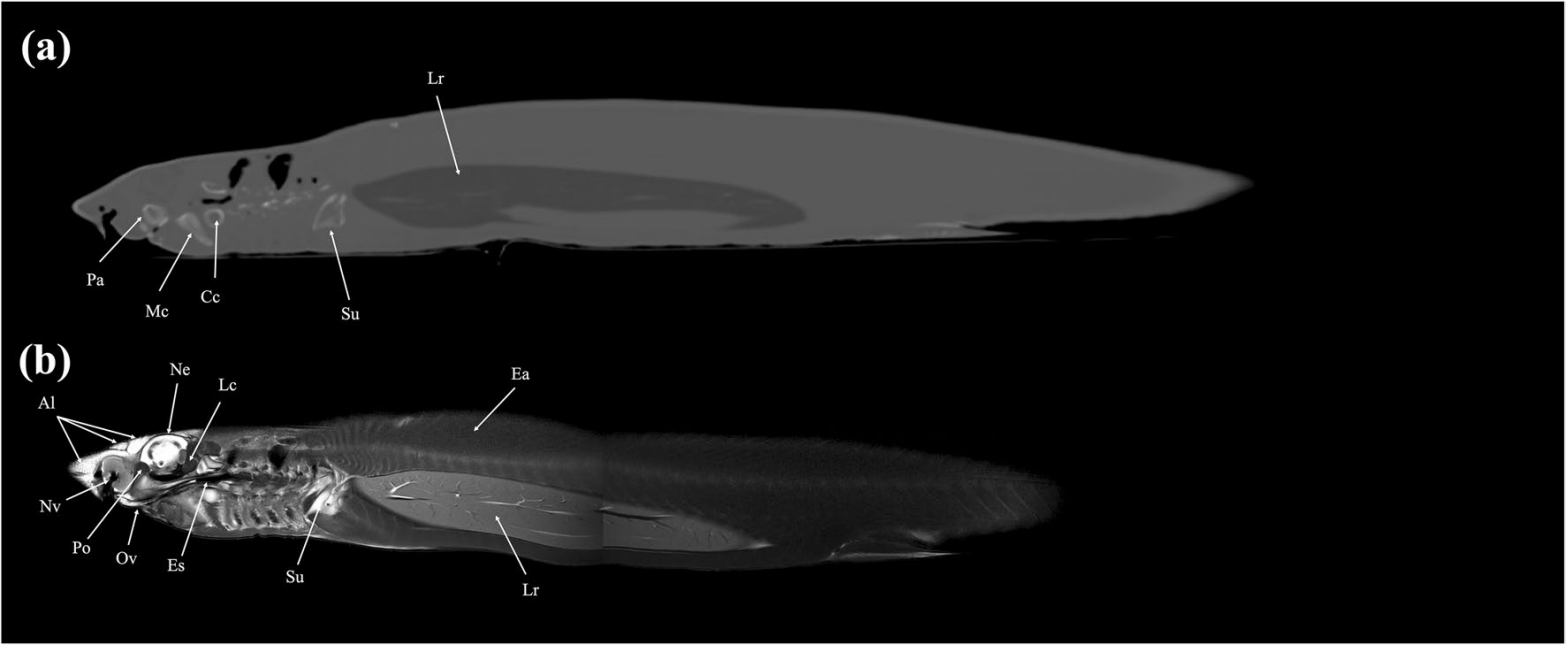
Sagittal (**a**) CT and (**b**) MR images of a banded houndshark (*Triakis scyllium*) at level 17 of Fig. 25. *Al* Ampullae of Lorenzini, *Am* Adductor mandibularis, *Ba* Basihyal cartilage, *Cc* Ceratohyal cartilage, *Es* Esophagus, *Hc* Hyomandibular canal, *Ic* Infraorbital canal, *Io* Inferior obliquus, *Lc* Levator cranium maxillae, *Le* Lens, *Mc* Meckel’s cartilage, *Mp* Metapterygium, *Mt* Mandibular teeth, *Ne* Neurocranium, *Ni* Nictitating fold, *Ol* Optic lobe, *Op* Optic nerve, *Ov* Oral cavity, *Pa* Palatoquadrate, *Po* Preorbitalis, *Pr* Posterior rectus, *Sc* Supraorbital canal, *Su* Scapular cartilage, *So* Superior obliquus, *Th* Thalamus, *Ty* Thyroid, *Vi* Vitreous humor.

**Figure 18:**
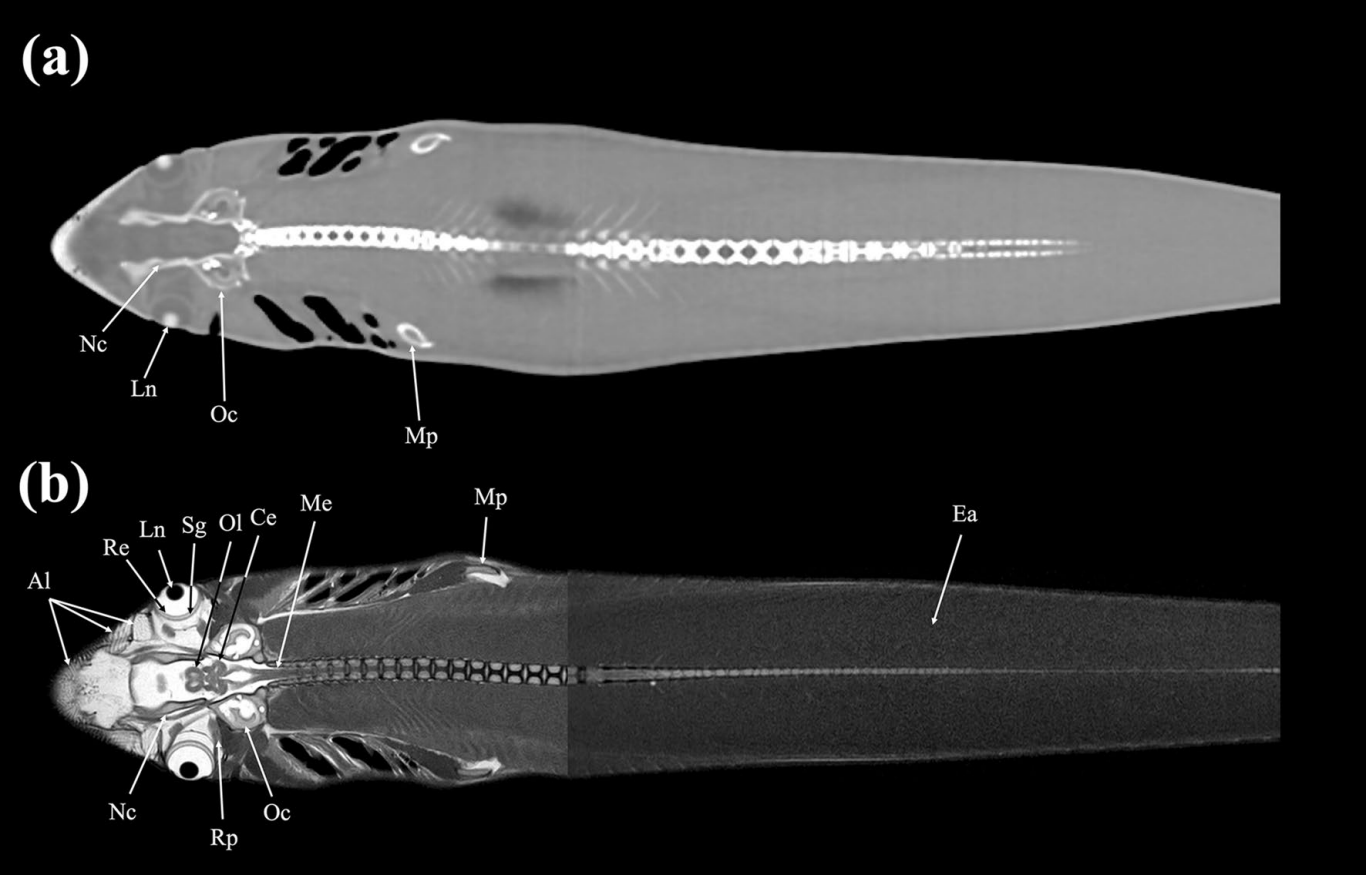
Dorsal (**a**) CT and (**b**) MR images of a banded houndshark (*Triakis scyllium*) at level 18 of Fig. 25. *Al* Ampullae of Lorenzini, *Ce* Cerebellum, *Ea* Epaxial, *Sg* Scleral cartilage, *Ln* Lens, *Me* Medulla oblongata, *Mp* Metapterygium, *Nc* Neurocranium, *Oc* Otic capsule, *Ol* Optic lobe, *Re* Retina, *Rp* Rectus posterior.

**Figure 19:**
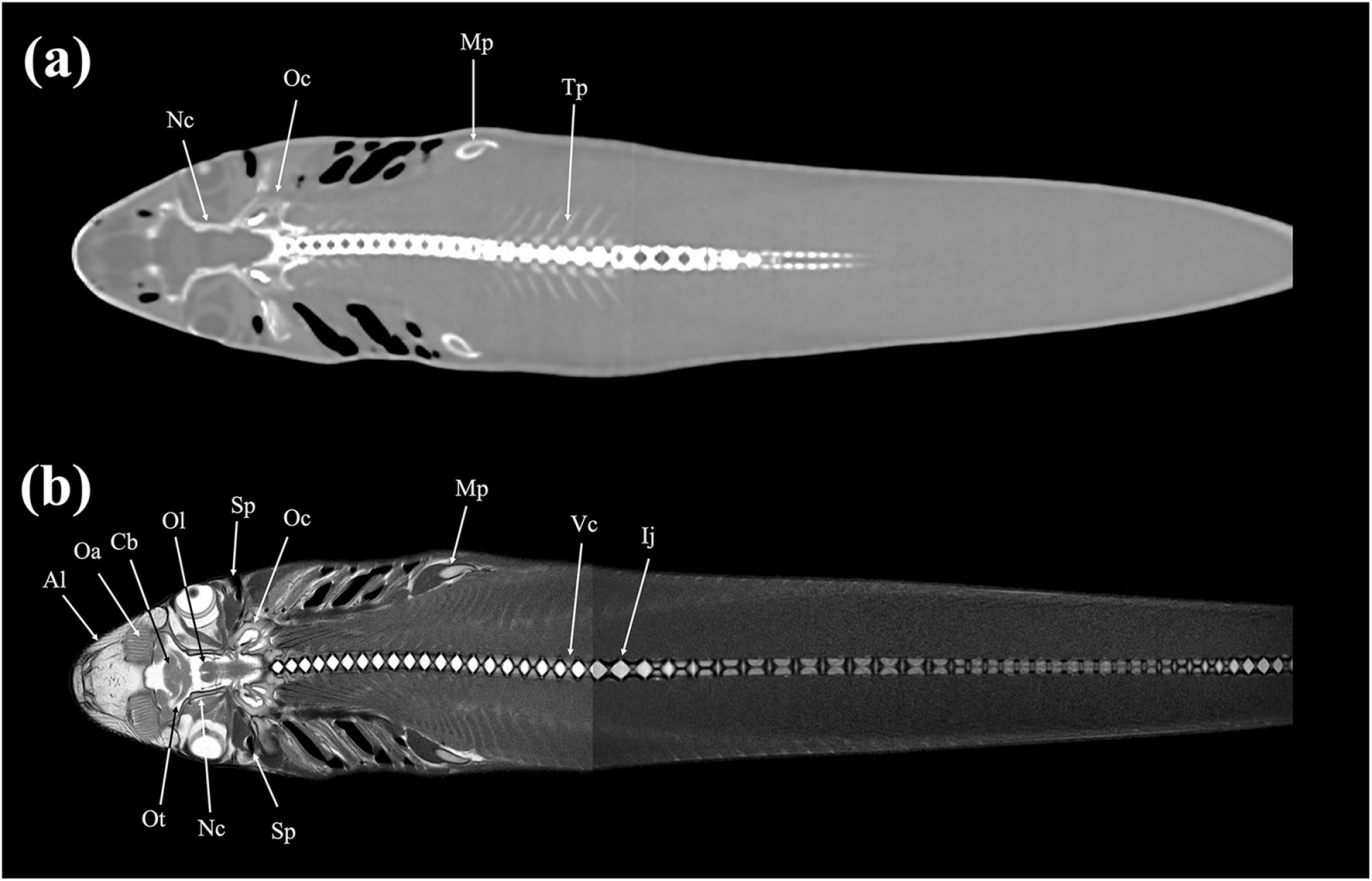
Dorsal (**a**) CT and (**b**) MR images of a banded houndshark (*Triakis scyllium*) at level 19 of Fig. 25. *Al* Ampullae of Lorenzini, *Cb* Cerebrum, *Ij* Intervertebral junction, *Mp* Metapterygium, *Nc* Neurocranium, *Oa* Olfactory lamellae, *Oc* Otic capsule, *Ol* Optic lobe, *Ot* Olfactory tract, *Sp* Spiracle, *Tp* Transverse process, *Vc* Vertebral centrum.

**Figure 20:**
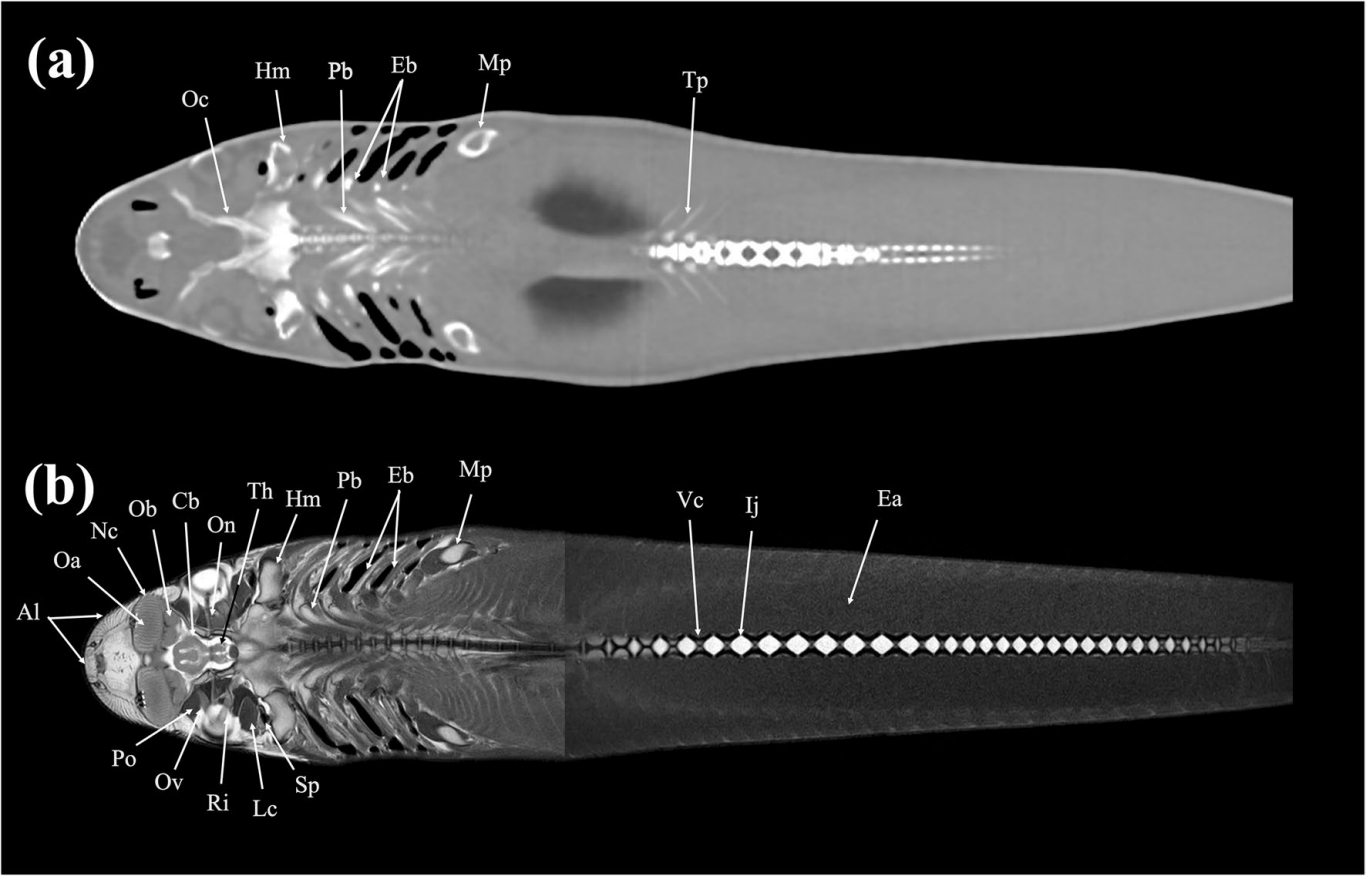
Dorsal (**a**) CT and (**b**) MR images of a banded houndshark (*Triakis scyllium*) at level 20 of Fig. 25. *Al* Ampullae of Lorenzini, *Cb* Cerebrum, *Ea* Epaxial, *Eb* Epibranchial, *Hm* Hyomandibular, *Ij* Intervertebral joint, *Lc* Levator cranium maxillae, *Mp* Metapterygium, *Nc* Nasal capsule, *Oa* Olfactory lamellae, *Ob* Olfactory bulb, *Oc* Optic capsule, *On* Optic nerve, *Ov* Obliquus ventralis, *Pb* Pharyngobranchial, *Po* Preorbitalis, *Ri* Rectus inferior, *Sp* Spiracle, *Th* Thalamus, *Vc* Vertebral centrum.

**Figure 21:**
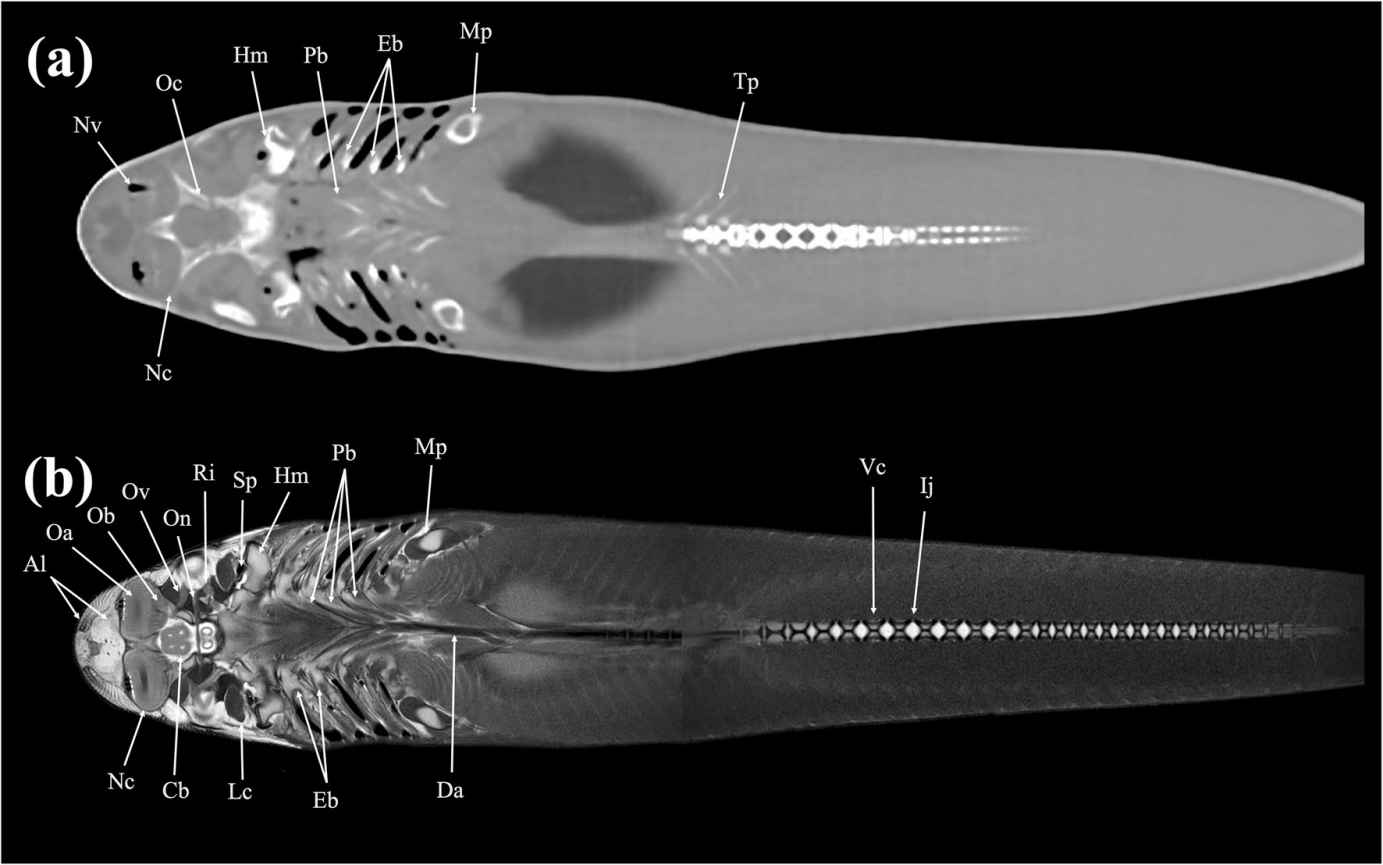
Dorsal (**a**) CT and (**b**) MR images of a banded houndshark (*Triakis scyllium*) at level 21 of Fig. 25. *Al* Ampullae of Lorenzini, *Cb* Cerebrum; *Da* Dorsal aorta, *Eb* Epibranchial, *Hm* Hyomandibular, *Ij* Intervertebral junction, *Lc* Levator cranium maxillae, *Mp* Metapterygium, *Nc* Nasal capsule, *Nv* Nasal cavity, *Oa* Olfactory lamellae, *Ob* Olfactory bulb, *Oc* Optic capsule, *On* Optic nerve, *Ov* Obliquus ventralis, *Pb* Pharyngobranchial, *Tp* Transverse process, *Ri* Rectus inferior, *Sp* Spiracle, *Vc* Vertebral centrum.

**Figure 22:**
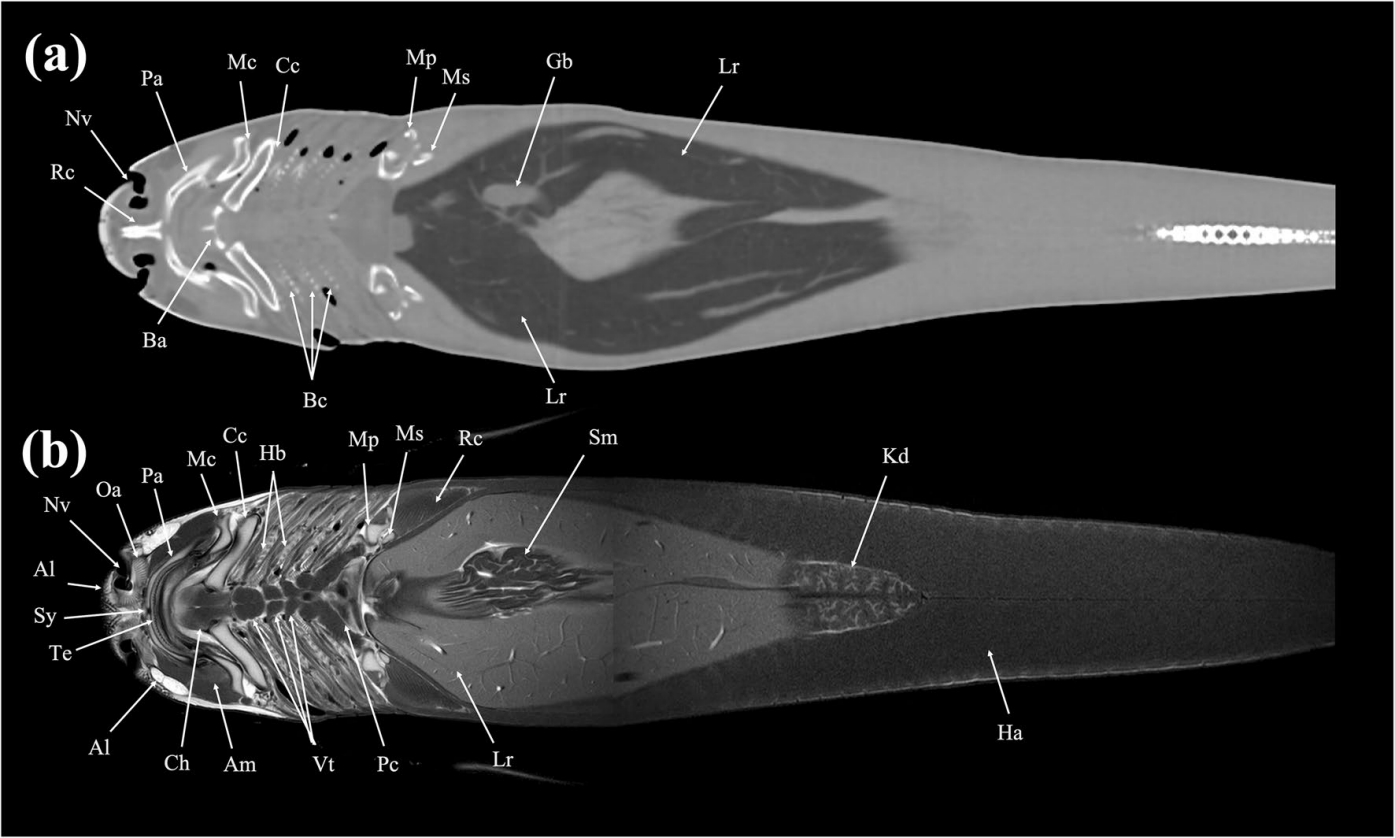
Dorsal (**a**) CT and (**b**) MR images of a banded houndshark (*Triakis scyllium*) at level 22 of Fig. 25. *Al* Ampullae of Lorenzini, *Am* Adductor mandibularis, *Ba* Basihyal cartilage, *Bc* Branchial cartilage, *Cc* Ceratohyal cartilage, *Ch* Coracohyoideus, *Ha* Hypaxial, *Hb* Hypobranchial, *Gb* Gall bladder, *Kd* Kidney, *Lr* Liver, *Mc* Meckel’s cartilage, *Ms* Mesopterygium, *Mp* Metapterygium, *Nv* Nasal cavity, *Oa* Olfactory lamellae, *Pa* Palatoquadrate, *Pc* Pericardial cavity, *Vt* Ventral constrictors, *Rc* Rostral cartilage, *Te* Teeth, *Sm* Stomach, *Sy* Symphysis.

**Figure 23:**
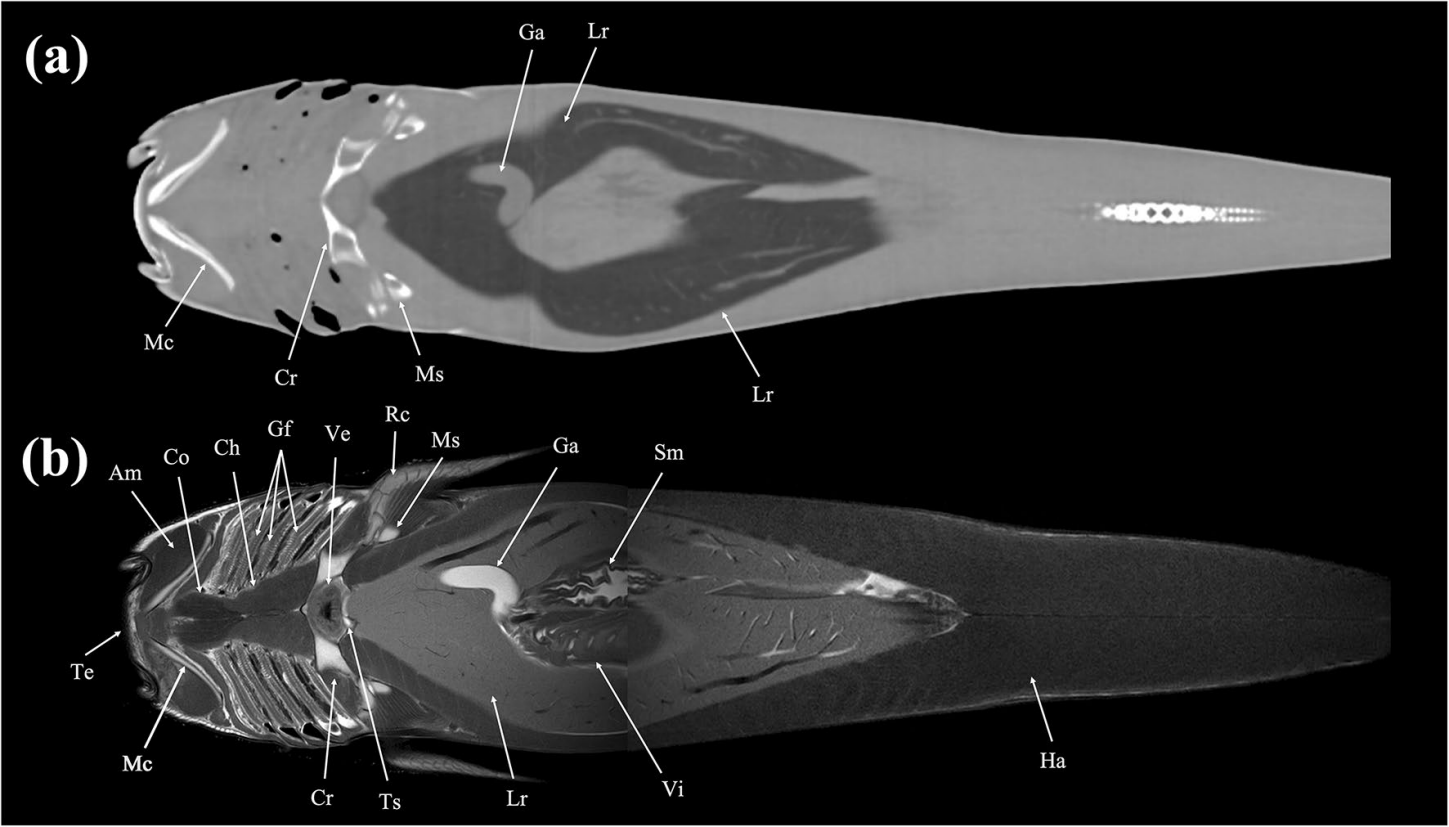
Dorsal (**a**) CT and (**b**) MR images of a banded houndshark (*Triakis scyllium*) at level 23 of Fig. 25. *Am* Adductor mandibularis, *Co* Coracomandibularis, *Cr* Coracoid bar, *Ch* Coracohyoideus, *Ga* Gall bladder, *Gf* Gill filament, *Ha* Hypaxial, *Lr* Liver, *Mc* Meckel’s cartilage, *Ms* Mesopterygium, *Rc* Radial cartilage, *Sm* Stomach, *Te* Teeth, *Ts* Transverse septum, *Ve* Ventricle, *Vi* Valvular intestine.

**Figure 24:**
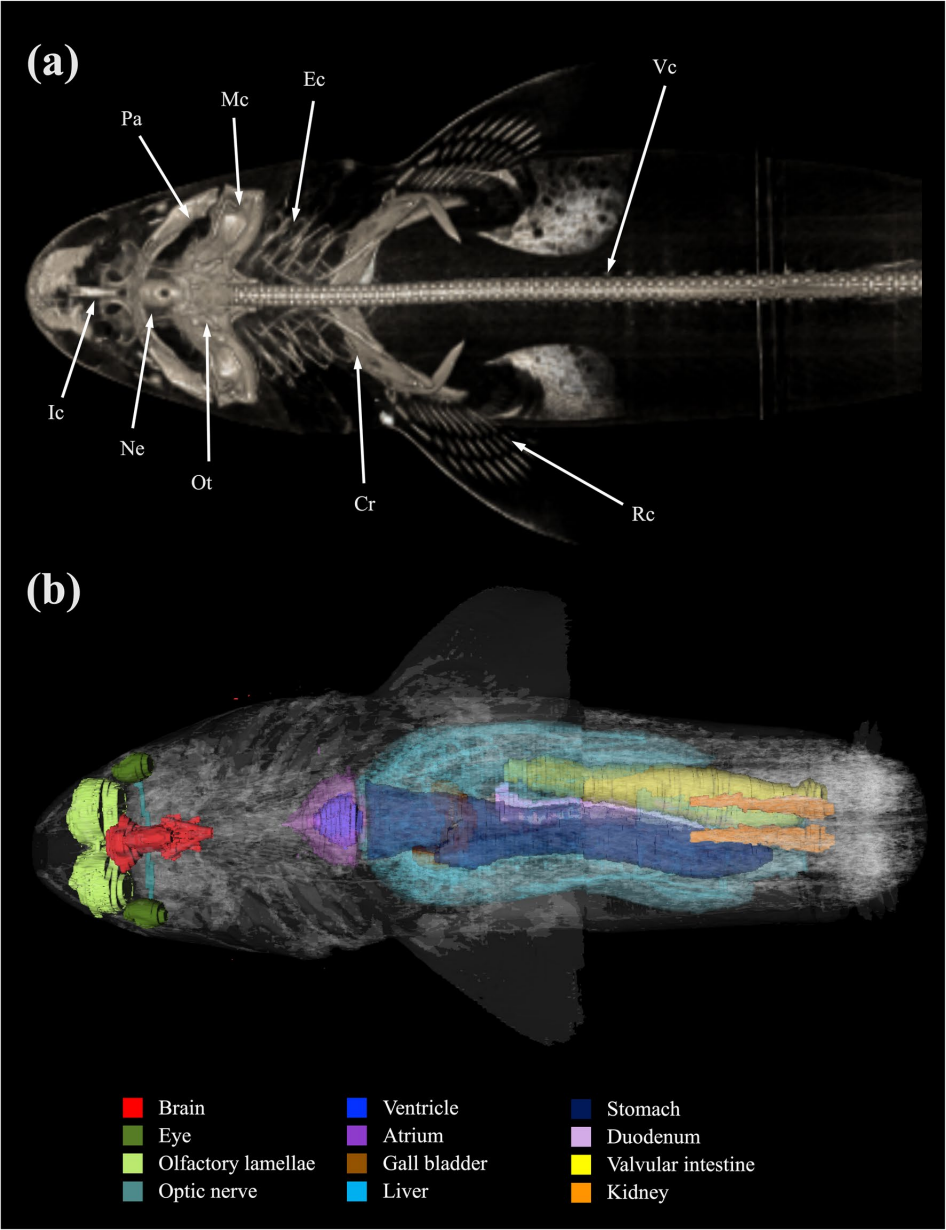
Three dimensional reconstructed images of (**a**) CT and (**b**) MRI showing major skeletal structures and organs respectively of a banded houndshark (*Triakis scyllium*). *Cr* Coracoid bar, *Ec* Epibranchial and ceratobranchial cartilage, *Ic* Internasal cartilage, *Mc* Meckel’s cartilage, *Ne* Neurocranium, *Ot* Otolith, *Pa* Palatoquadrate, *Rc* Radial cartilage, *Vc* Vertebral centrum.

### Transverse images

Figures 1, 2, 3, 4, 5, 6, 7, 8, 9, 10, 11, 12, 13, 14.

### Sagittal images

Figures 15, 16, 17.

### Dorsal images

Figures 18, 19, 20, 21, 22, 23.

## Discussion

The MRI signal intensity is proportional to the hydrogen density of the structures, ensuring excellent definition of soft tissues, organs, and cavitary structures in banded houndsharks. These structures included major muscles; cardiovascular system structures, including the atrium, ventricle, and major arteries and veins; digestive system structures, including the stomach, gall bladder, and valvular intestine; excretory system structures, including the kidney; nervous system structures, including the cerebrum, optic lobe, and thalamus. Due to the absence of hydrogen atoms, most of the cartilaginous skeletons in banded houndsharks did not provide sufficient magnetic resonance signal and showed a hypointense (black/dark grey) pattern. Nonetheless, some cartilaginous structures, such as the jaws and coracoid bar, could still be observed due to the contrast between the cartilage and the adjacent soft tissues.

In contrast to MRI, excellent discrimination of cartilaginous structures was evident in the CT images. The higher electron density of cartilages caused increased attenuation compared to soft tissue, making them appear more whitish in the CT images. The cartilage margins were better defined by means of CT than cryosections or MRI, particularly in assessing joint structures. Soft tissues were vaguely distinguished using CT examinations due to their similar attenuation properties. Nevertheless, attenuation differences between lipid-rich tissues (e.g., liver) and muscles (e.g., stomach and valvular intestine) were shown in varying shades of grey allowing definition of some organs in the visceral cavity.

CT and MRI offer considerable advantages over traditional radiographic approaches on identification of the anatomical structures of sharks. In traditional radiography, images are presented as superimposed two-dimensional projections of three-dimensional structures, which result in limitation to exhibit high conspicuity. The tomographic nature of CT and MRI allows organs to be examined in thin sections, eliminating superimposition of overlying structures that may hinder the specific interpretation of anatomical structures.

Another major advantage of CT and MRI is the ability to reformat the datasets in any imaging plane or as 3D projections, allowing better understanding of the spatial relationships of anatomical structures in sharks. These techniques have added detail of the anatomical structures and can be used as an anatomic reference for imaging studies of the banded houndshark and other shark species. It is recommended to reduce CT/MRI slice thickness as much as possible in order to achieve excellent quality and high detail of 3D reconstructed images, since increasing slice thicknesses may require more interpolation between slices when rendering 3D images, which results in a loss of resolution^40^.

Due to their important phylogenetic position among extant vertebrates, sharks are an invaluable group in evolutionary developmental biology studies. However, the presence of high levels of urea accelerates the decomposition of shark flesh^41^. Moreover, because of its cartilaginous nature, shark skeleton has a high tendency to warp, crack, and shrink during specimen preparation^42^. These factors represent major difficulties in preserving anatomical data from sharks. In the present study, CT and MRI were used to identify and document soft tissues and cartilaginous structures in banded houndsharks. The non-invasive nature of CT and MRI prevented the irretrievable destruction of specimens during traditional dissection. The spatial relationships among organs, soft tissues, and cartilage can be selectively observed in situ in their natural locations^43,44^. After examinations, all images are permanently recorded in the Digital Imaging and Communications in Medicine (DICOM) format and could be recalled at will. The images are digitally transferable, which can also facilitate discussion and opinion sharing among professionals worldwide even if the specimens cannot be physically provided.

Banded houndshark is a relatively small-sized shark species that can be adequately fit into the bore of medical CT and MRI used in the present study. However, to the best of the authors’ knowledge, only the head of large-sized shark species has been subjected to imaging examination possibly because of limited gantry size^45–47^. Sharks with body girth larger than the maximum diameter of field of view (FOV) of medical CT and MRI may also induce out-of-field artifacts, anatomical structures that lies beyond the FOV may be truncated. With the rapid advancement of medical imaging technologies, wide bore CT and MRI have become available for large animals^48–50^. Although the current availability of wide bore CT and MRI scanners are limited and the set up cost is high, the use of wide bore CT and MRI may offer a feasible alternative for imaging examination of large-sized shark species in future.

## Conclusions

The illustrations in the present study are the first to provide a comprehensive atlas, using CT and MRI, to evaluate the anatomical structures of the banded houndshark. Both imaging modalities provide good contrast for the anatomical structures of the banded houndshark. With the rapid advancement and increasing availability of medical imaging technologies, CT and MRI are enhancing our knowledge of the anatomy of sharks together with dissected and necropsy materials. The findings of the present study could be considered as a useful reference of normal tomographic anatomy of sharks and used for the interpretation of normal and pathological imaging studies of sharks in future.

## Material and methods

### Animals

Three banded houndsharks were obtained commercially from Seoul, the Republic of Korea. The banded houndsharks were 1 male (total body length (TBL): 90 cm, age: estimated as 3.4 years) and 2 females (TBL: 91 cm, age: estimated as 3.3 years; TBL: 102 cm, age: estimated as 4.1 years) with body lengths ranging from 90 to 102 cm. Ages of the sharks were estimated based on their TBL and it was judged that the sharks were about to be sexually mature^51^. All animals were selected by a veterinarian (SWK) based on the lack of external evidence of trauma and disease. The banded houndsharks were euthanized via tricaine methanesulfonate overdose (500 ppm) right before imaging examinations in order to minimize post-mortem changes.

### Magnetic resonance imaging (MRI)

MRI examinations were performed using a 3-T (T) MRI scanner (Achieva, Philips Healthcare, The Netherlands). A head coil was used to receive the signal using fast spin echo sequences in the T2-weighted (T2W) mode, in which signals from loosely bound protons (e.g., fluids) were enhanced:

1. Transverse images were obtained with the following parameters: echo time (TE), 90 ms; repetition time (TR), 4920.8 ms; pixel bandwidth, 180 Hz; flip angle, 90°, and FOV, 123 × 123 mm with reconstructed spacing, 0.17 × 0.17 mm. Slice thickness was 2 mm.
2. Sagittal images were obtained with the following parameters: TE, 90 ms; TR, 4870.9 ms; pixel bandwidth, 215 Hz; flip angle, 90°, and FOV, 76 × 76 mm with reconstructed spacing, 0.20 × 0.20 mm. Slice thickness was 3 mm.
3. Dorsal images were obtained with the following parameters: TE, 70 ms; TR, 3865.9 ms; pixel bandwidth, 236 Hz; flip angle, 90°, and FOV, 81 × 81 mm with reconstructed spacing, 0.23 × 0.23 mm. Slice thickness was 1.5 mm.

### Computed tomography (CT)

CT images were acquired with a 64-detector row spiral CT scanner (Aquilion, Toshiba Medical Systems, Nasu, Japan). The CT examination was performed at 120 kV and 80–100 mA with a 1-mm slice thickness. The scan field of view (sFOV) ranged from 20.3 to 30.2 cm. The transverse image datasets were acquired using the soft tissue and bone algorithm. CT images in the dorsal and sagittal dimensions were obtained using the multiplanar reconstruction (MPR) function. MRI and CT images were assessed using open-source DICOM viewer software (Horos Project, version 3.3.6; www.horosproject.org).

### Cryosectioning

**Figure 25:**
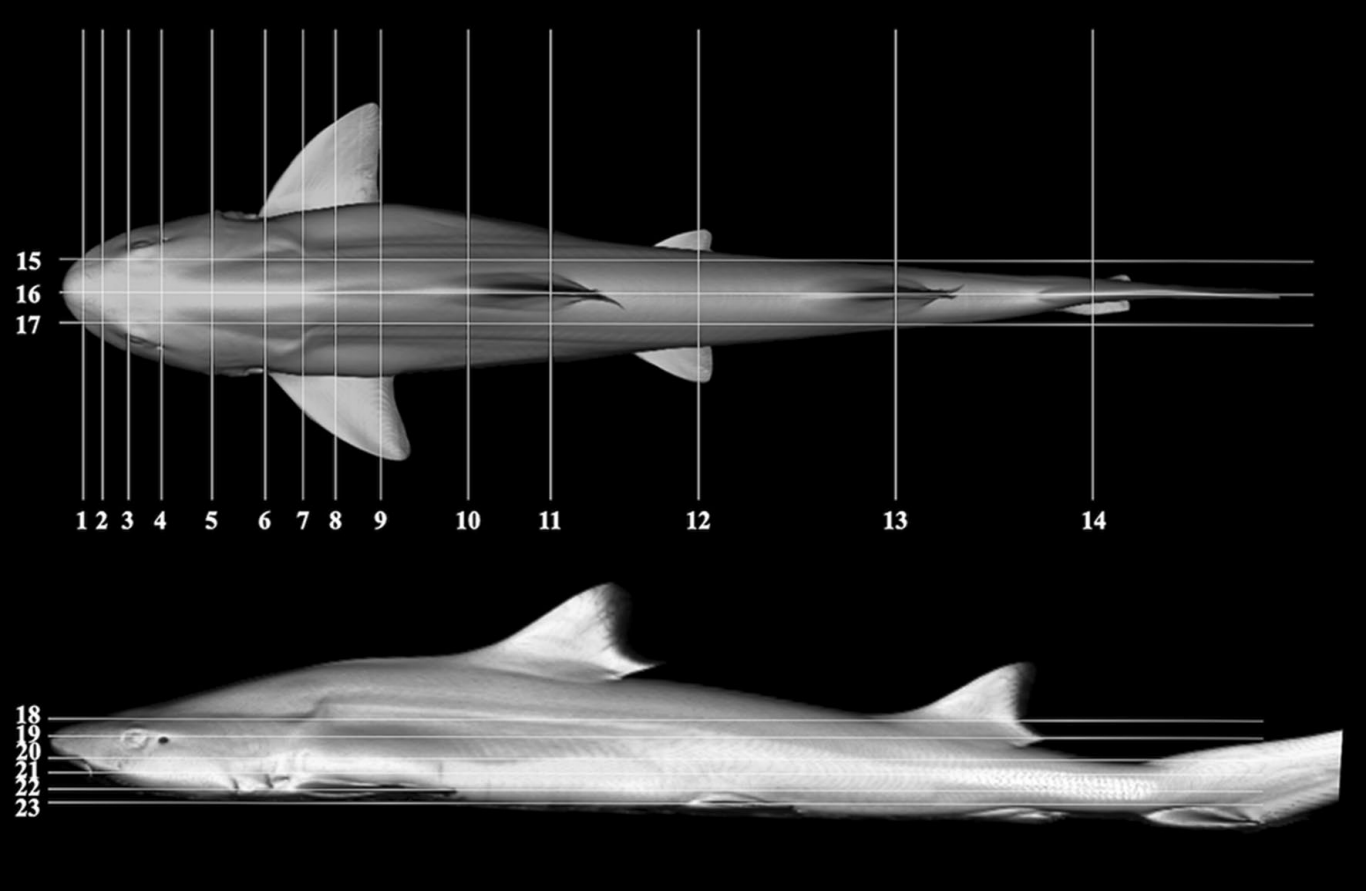
Three-dimensional surface reconstructions of a banded houndshark (*Triakis scyllium*) from computed tomography (CT) scans, indicating approximate levels of transverse, sagittal, and dorsal sections corresponding to Figs. 1, 2, 3, 4, 5, 6, 7, 8, 9, 10, 11, 12, 13, 14, Figs. 15, 16, 17, and Figs. 18, 19, 20, 21, 22, 23, respectively. Slices 1–4 are approximately 2 cm intervals, slices 4–9 are approximately 5 cm intervals, slices 9–11 are approximately 6 cm intervals, slices 11–14 are approximately 12 cm intervals, slices 15–17 are approximately 3 cm intervals, and slices 18–23 are approximately 1 cm intervals.

All specimens were frozen (− 22 °C) in the ventral recumbency position until cryosectioning could be carried out. The banded houndsharks were sectioned using an electrical band saw along the transverse plane at approximately 1-cm intervals. Slices were numbered, cleaned, and photographed on both sides. Fourteen transverse section levels were selected. For each section level, the corresponding transverse MRI and CT images were chosen to identify the best anatomical correlation. Lines corresponding to these levels were superimposed on three-dimensional reconstructed images of an banded houndshark (Fig. 25).

### Three-dimensional (3D) reconstruction

3D reconstructions of the skeletons were made on acquired CT data sets using intensity-based segmentation methods of the inbuilt software of 64-detector row spiral CT scanner (Aquilion, Toshiba Medical Systems, Nasu, Japan). The reconstructed skeletons were visualised after excluding remaining residual soft tissue structures. 3D reconstructions of major organs were made on MRI data sets using intensity-based and manual segmentation methods of an open-source DICOM viewer—InVesalius 3.0 (CTI—Center for Information and Technology Renato Archer, Campinas, São Paulo, Brazil).

### Ethical approval

The study was approved by the Seoul National University Institutional Animal Care and Use Committee (approval number: SNU-190925-2). All experiments were performed in accordance with the IACUC guidelines and regulations. This study was also performed in accordance with the Animal Research: Reporting of In Vivo Experiments (ARRIVE) guidelines: to reflect the scarcity of banded houndshark over 90 cm TBL in the fish markets of the Republic of Korea, sample size was decided to three; to minimize the potential confounders, both male and female sharks were used; to show the developmental stages, TBL and estimated age of each shark were provided.

## Supplementary Information

Supplementary Information 1.

Supplementary Information 2.

## Data Availability

All data generated or analyzed during this study are included in this published article.
