# Supplementary material for: Cross-sectional anatomy, computed tomography, and magnetic resonance imaging of the banded houndshark (Triakis scyllium)
Source: Sci Rep. 2021 Jan 13;11:1165. doi: 10.1038/s41598-020-80823-y (PMC7806778; doi:10.1038/s41598-020-80823-y)
Supplement: Supplementary file 1 — Supplementary Information 1. [file 41598_2020_80823_MOESM1_ESM.docx]

**Cross-sectional anatomy, computed tomography, and magnetic resonance imaging of the banded houndshark (*Triakis scyllium*)**

**[Supplementary information]**

Sang Wha Kim^a‡^, Adams Hei Long Yuen^a‡^, Cherry Tsz Ching Poon^b^, Joon Oh Hwang^c^, Chang Jun Lee^c^, Moon-Kwan Oh^d^, Ki Tae Kim^a^, Hyoun Joong Kim^a^, Sib Sankar Giri^a^, Sang Guen Kim^a^, Jun Kwon^a^, Sung Bin Lee^a^, Min Cheol Choi^a^, and Se Chang Park^a,*^

^a^College of Veterinary Medicine and the Research Institute for Veterinary Science, Seoul National University, Seoul 08826, Republic of Korea

^b^Department of Surgery, Queen Mary Hospital, Pokfulam, Hong Kong Special Administrative Region, China

^c^Hyemin Animal Hospital, Seoul 06239, Republic of Korea

^d^HDX corporation, Seoul 03162, Republic of Korea

* Corresponding author

*E-mail address*: [parksec@snu.ac.kr](mailto:parksec@snu.ac.kr) (S.C. Park)

^‡^ Sang Wha KIM and Adams Hei Long YUEN contributed equally to this study.

**[Supplementary information title page]**

**Supplementary Video S1.** Transverse MRI images of *Triakis scyllium*.

**Supplementary Video S2.** Transverse CT images of *Triakis scyllium*.
